# Biofilm removal effect of diatom complex on 3D printed denture base resin

**DOI:** 10.1038/s41598-024-54408-y

**Published:** 2024-02-19

**Authors:** Sung-sil Choi, Joo Hun Lee, Hyunjoon Kong, Eun-Jin Park

**Affiliations:** 1 Department of Dental Laboratory Technology, Graduate School of Clinical Dentistry, Ewha Womans University, Seoul, 07985 Republic of Korea; 2 Department of Chemical and Biomolecular Engineering, University of Illinois at Urbana- Champaign, Urbana, 61801 USA; 3 Department of Prosthodontics, College of Medicine, Ewha Womans University, 25, Magokdong-ro 2-gil, Gangseo-gu, Seoul, 07804 Republic of Korea

**Keywords:** Diatom complex, Active micro-locomotion, Biofilm, Denture, Health care, Medical research

## Abstract

For patients who have difficulty in mechanical cleaning of dental appliances, a denture cleaner that can remove biofilm with dense extracellular polymeric substances is needed. The purpose of this study is to evaluate the efficacy of diatom complex with active micro-locomotion for removing biofilms from 3D printed dentures. The diatom complex, which is made by doping MnO_2_ nanosheets on diatom biosilica, is mixed with H_2_O_2_ to generate fine air bubbles continuously. Denture base resin specimens were 3D printed in a roof shape, and *Pseudomonas aeruginosa* (10^7^ CFU/mL) was cultured on those for biofilm formation. Cleaning solutions of phosphate-buffered saline (negative control, NC), 3% H_2_O_2_ with peracetic acid (positive control, PC), denture cleanser tablet (DCT), 3% H_2_O_2_ with 2 mg/mL diatom complex M (Melosira, DM), 3% H_2_O_2_ with 2 mg/mL diatom complex A (Aulacoseira, DA), and DCT with 2 mg/mL DM were prepared and applied. To assess the efficacy of biofilm removal quantitatively, absorbance after cleaning was measured. To evaluate the stability of long-term use, surface roughness, ΔE, surface micro-hardness, and flexural strength of the 3D printed dentures were measured before and after cleaning. Cytotoxicity was evaluated using Cell Counting Kit-8. All statistical analyses were conducted using SPSS for Windows with one-way ANOVA, followed by Scheffe’s test as a post hoc (*p* < 0.05). The group treated with 3% H_2_O_2_ with DA demonstrated the lowest absorbance value, followed by the groups treated with 3% H_2_O_2_ with DM, PC, DCT, DCT + DM, and finally NC. As a result of Scheffe’s test to evaluate the significance of difference between the mean values of each group, statistically significant differences were shown in all groups based on the NC group. The DA and DM groups showed the largest mean difference though there was no significant difference between the two groups. Regarding the evaluation of physical and mechanical properties of the denture base resin, no statistically significant differences were observed before and after cleaning. In the cytotoxicity test, the relative cell count was over 70%, reflecting an absence of cytotoxicity. The diatom complex utilizing active micro-locomotion has effective biofilm removal ability and has a minimal effect in physical and mechanical properties of the substrate with no cytotoxicity.

## Background

In 2018, Kong et al. introduced a diatom complex made by doping manganese oxide nanosheets on porous diatom biosilica, which was mixed with hydrogen peroxide to continuously generate microbubbles through the decomposition reaction of H_2_O_2_ by MnO_2_. The bubble generation caused self-locomotion of the diatom particles and actively ruptured the EPS matrix of biofilm^1,2^. This diatom complex demonstrated effectiveness in removing biofilm formed by Gram-negative *Escherichia coli* on a micro-grooved polydimethylsiloxane substrate compared to a group treated with only 3% H_2_O_2_^1^. Subsequently, the diatom complex was introduced for use in the field of dentistry. Specifically, it was shown that MnO_2_-diatoms were more effective in removing *Streptococcus mutans* and *Porphyromonas gingivalis* cultured on various dental prosthetic materials compared to chlorhexidine gluconate or hydrogen peroxide^3^. The further findings confirmed the efficiency, stability, and biocompatibility of the diatom complex for even treating peri-implantitis.^4^

Many societies are currently experiencing a rapid transition toa super-aged society as life expectancy increases. Alongside this demographic shift, there is a notable increase in the number of denture users. However, denture wearers' denture cleanliness and oral hygiene were generally found to be poor^5,6^. If the denture surface is worn down due to improper dental cleaning, various bacteria such as *Candida albicans*, *Staphylococcus aureus*, *Streptococcus mutans*, *Pseudomonas aeruginosa*, *and Enterobacter amnigenus* can grow on the denture base resin^7,8^. Poor denture hygiene puts patients at risk of denture stomatitis, oral malodor, dental caries, periodontitis, and systemic infections associated with oral bacteria^9,10^. The oral health of the elderly is closely related to the overall health and quality of life^11^, so it is very important to ensure dental cleanliness for elderly patients. In particular, elderly patients with compromised immune systems must ensure that bacterial plaque, tartar, and external stains on the denture surface are removed through appropriate cleaning methods.^12^.

Denture hygiene maintenance involves either mechanical or chemical, or a combination of both, methods^13,14^. Mechanical cleaning through brushing is the most commonly utilized and effective method among denture wearers^15^, but is often difficult for older and physically disabled denture wearers^16^. Meanwhile, a chemical method includes soaking dentures in water using a denture cleaner, which is effective in removing microorganisms and stains on the surface of dentures^17,18^. However, for biofilm that has strongly adhered over an extended period of time, it remains challenging to remove it solely through the use of a chemical denture cleaner^12,19,20^. Therefore, complementing the use of a chemical denture cleaner with mechanical brushing would be more effective in biofilm cleaning than solely using a chemical or mechanical method.

With the advancement of CAD/CAM (Computer-Aided Design and Computer-Aided Manufacturing) technology, 3D printed dentures are rapidly developing in the field of dentistry. Dentures produced through 3D printing have the advantages of simplicity, low porosity, reduced number of patient visits, and easy reuse with saved design files compared to heat polymerization denture production technology^21^. Furthermore, 3D-printed dentures are built layer by layer without material waste, which is more economical than the subtractive technique, milling^22^. Appropriate denture resin materials should exhibit no physical and mechanical property changes^23^, but according to a study comparing the surface roughness changes of denture materials fabricated using three different techniques (conventional heat-curing, CAD/CAM, and 3D printing) after cleaning with denture cleaners, the greatest changes were observed in the 3D-printed denture materials^24^. Another study reported changes in hardness and color stability of 3D-printed denture materials after cleaning with denture cleaners^25,26^. Although many studies have been conducted on the effect of using denture cleaners on conventional heat-curing resin materials^27–29^, there is a lack of research on the effects of denture cleaners on 3D printed denture materials. Since denture cleaners are soaked and used every day, it is essential to consider the effects of long-term use. Additionally, considering that dentures are used in the oral cavity, biocompatibility evaluation is essential.

This study aims to evaluate the elimination of biofilm formed on 3D printed denture base resin by comparing two types of diatom complexes with DCT and peracetic acid, which are commonly recommended to disinfect dental equipment. In addition, changes in physical and mechanical properties and the cytotoxicity of denture resin material before and after cleansing were assessed. The first null hypothesis is that the biofilm removal ability of diatom complex is not different from that of denture cleanser tablet, and the second null hypothesis is that the diatom complex does not show any difference in the physical and mechanical properties and cytotoxicity of the denture base resin before and after cleansing compared with DCT.

## Methods

**Figure 1:**
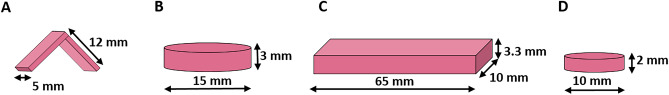
Shape of the specimens used in each experiment. (**A**) Evaluation of biofilm removal ability. (**B**) Color and micro-hardness. (**C**) Surface roughness and flexural strength. (**D**) Cytotoxicity test.

The 3D printed denture base resin specimen to evaluate the biofilm removal ability was designed in the shape of a roof (12 x 5 x 12 mm) to mimic the curved inner surface of the denture, which is difficult to clean. Using denture base resin (Tera Harz TFD-23-5; Graphs), a total of 36 specimens, 6 for each group, were fabricated. For the experiment on the physical and mechanical properties, a disc shape (15 × 3 mm) was designed to measure color and micro-hardness, and a plate shape (65 × 10 × 3.3 mm) was designed to measure surface roughness and flexural strength. A total of 40 specimens, 10 for each group, were fabricated using a definitive 3D printed denture base resin material (NextDent Base; Vertex Dental). In addition, for the cytotoxicity test, 40-disc shaped (10 × 2 mm) specimens were fabricated. 10 were digitally printed using permanent denture resin material (NextDent Base; Vertex Dental) and 30 were fabricated according to the manufacturer's instructions using thermal polymerization resin (SR Ivocap; Ivoclar) via a conventional method of processing dentures. The specimen shape designed for each experiment is shown in Fig. 1, while Table 1 shows the physical properties of the 3D printed denture base resins used. The data of each denture base resin specimen were extracted as a standard tessellation language file and printed using a 3D printer (T7L-130; Veltz). Post-curing was performed for 20 min using a UV curing machine (LC-3D print Box; Vertex Dental) to increase the degree of polymerization.

**Table 1:** Mechanical properties of the denture base resins.

Mechanical property	Tera Harz TFD-23-5	NextDent base
Hardness shore D	≥ 80	80–90
Flexural strength (MPa)	≥ 110	80–95
Flexural modulus (MPa)	≥ 2800	2000–2400
Shade	Magenta	Various shades of pink

**Figure 2:**
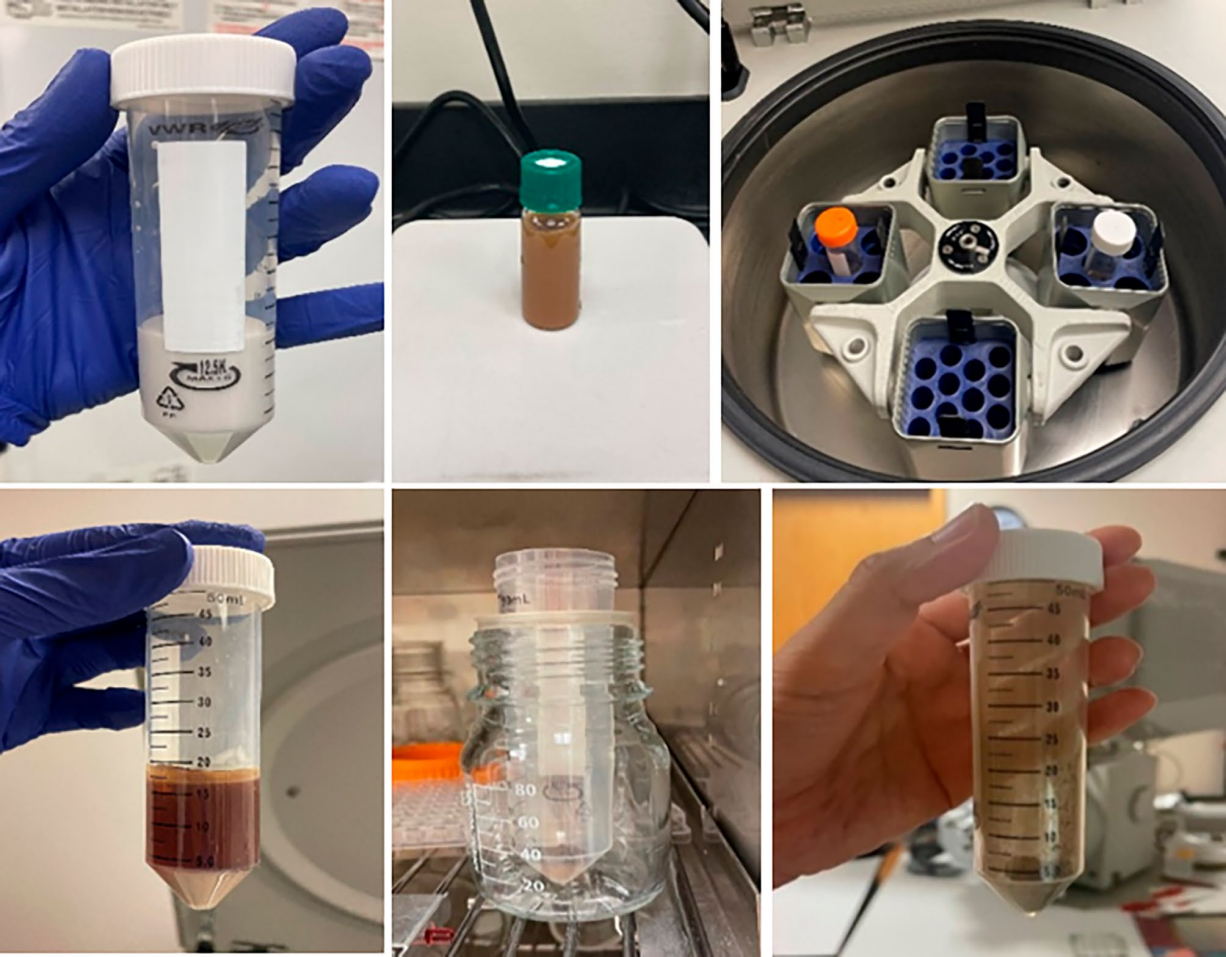
Fabrication process of diatom complex *Melosira.*

Diatom complex was made from seawater diatoms (*Melosira nummuloides*; JDK Bio Co.), and the process is shown in Fig. 2. One gram of *Melosira* was mixed with 15 ml of deionized water and 3 ml was extracted, followed by the addition of 15 ml of sugar and stirring for 10 min. Then, centrifugation was performed at 4000 rpm for 8 min, after which the supernatant liquid was discarded and new deionized water was added to the remaining particles, followed by centrifugation at 4000 rpm for 4 min. Then, 20 ml of deionized water and 0.316 g of KMnO_4_ were added to the obtained diatom particles, followed by stirring for 24 h. Then, the particles were collected by centrifugation at 1000 rpm for 5 min, washed with deionized water and dried in an oven at 60 °C for 24 h, resulting in the MnO_2_-doped DM. DA (provided from University of Illinois) used in a previous study was included in this work for comparison. The morphology and porosity of the DM were observed using a scanning electron microscope (QUANTA 450; FEI) at 5 kV. The motion of DM in the 3% H_2_O_2_ solution was observed using an optical microscope (Leica S6D; Leica, Wetzlar). A video was taken of the reaction after mixing 3% H_2_O_2_ and 2 mg/ml DM on a glass plate.

The *P. aeruginosa* (ATCC 15442) strain was cultured overnight at 37 °C on a Trypticase Soy Agar Plate (BD BBL), and then three to four colonies were mixed with 200 mL of Tryptic Soy Broth (TSB) (BD Bacto) and cultured overnight. New TSB was mixed with the original bacterial suspension at a ratio of 3:1 to form a new bacterial cell suspension (1000 µL) and it was dispensed into a 24-well culture plate. The roof shaped denture base resin specimens were placed and incubated for 24 h in a 37 °C incubator (100A; Blue M). After 24 h, the denture base resins were washed with phosphate-buffered saline (PBS) and a freshly mixed bacterial cell suspension was added. This process was repeated three times to form a biofilm for a total of 72 h. After incubating the biofilm, it was washed with 1 ml of PBS solution and placed in a new 24-well culture plate.

The denture base resin cultured with biofilm was divided into the following six groups and treated with 1 mL of each cleansing solution for 10 min. (1) PBS (NC), (2) 3% H_2_O_2_ with peracetic acid (PC), (3) DCT (Polident 5 min Quick Plus; GlaxoSmithKline), (4) 3% H_2_O_2_ with 2 mg/mL DM, (5) 3% H_2_O_2_ with 2 mg/mL DA, and (6) DCT with 2 mg/mL DM. For the groups with mixed cleansing solution, two people dispensed each solution at the same time to prevent errors due to time difference. After 10 min of treatment, the denture base resins were rinsed with PBS to remove residue and placed on a new 24-well culture plate.

**Figure 3:**
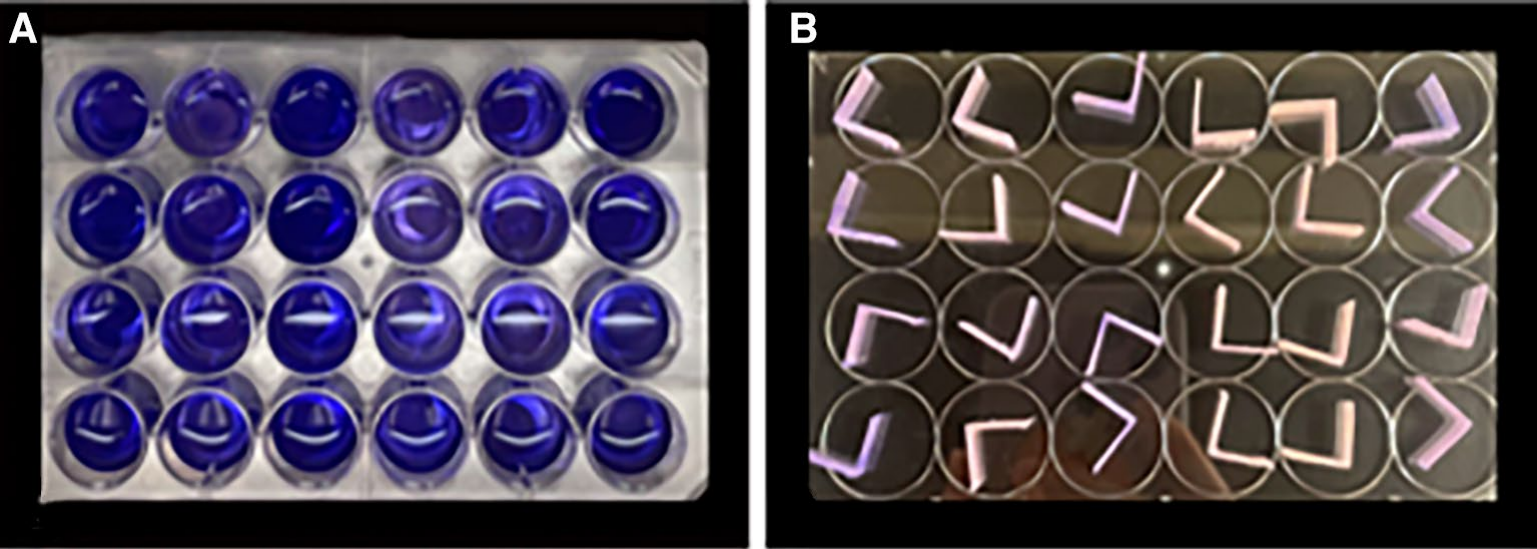
Crystal violet staining on 24-well culture plate. (**A**) Crystal violet solution was added for 30 min. (**B**) Specimens that had completed the crystal violet staining process**.**

Crystal violet staining was performed to quantitatively assess the effectiveness of biofilm removal. After each treatment, 1 ml of 0.1wt% crystal violet solution was added to the denture base resins placed on a 24-well culture plate and the remaining bacterial cells were stained for 30 min. The sample was then rinsed three times with PBS solution, moved to a new 24-well culture plate, and immersed in 95% ethanol to dissolve crystal violet within the biofilm. The absorbance of the dissolved solution was measured with an absorbance microplate reader (Infinite 200 Pro; Tecan) at a wavelength of 550 nm (Fig. 3). Immunostaining of the proteins in the biofilm was conducted by incubating the denture base resins with biofilms in 1 mL of 0.1 M NaHCO_3_ buffer (pH 9.2) containing 1 mg of fluorescein isothiocyanate (FITC, Sigma-Aldrich) for 1 h at room temperature. Polysaccharides in the biofilm were consecutively labeled by incubating the resins in the same buffer containing 250 µL of concanavalin A, tetramethylrhodamine (ConA-TMR, Sigma-Aldrich) for 2 h at room temperature. FITC labels the nuclei of the bacterial cells inside the biofilm while ConA-TMR labels the polysaccharides comprising most of the EPS inside the biofilm. Next, the samples were mounted on glass slides and observed with a confocal laser scanning microscope (LSM 700; Zeiss) to visualize the composition of the remaining biofilm.

**Figure 4:**
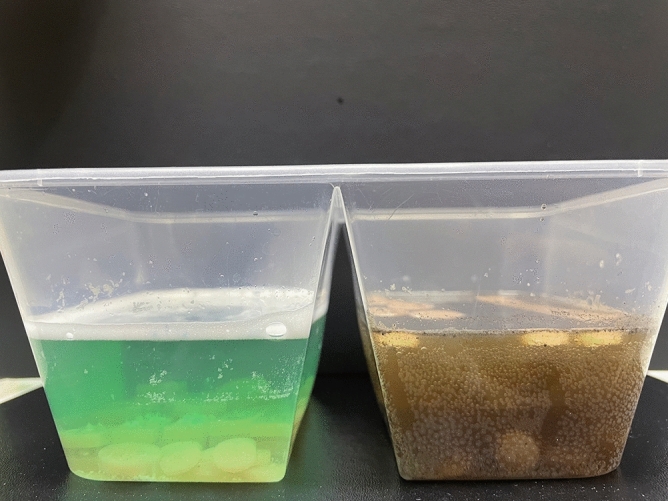
Denture cleansing tablet (left) and Diatom complex Melosira (right) immersion process

DM and DCT effects on the physical and mechanical properties of denture base resin were analyzed by simulating a condition of 5 years of daily cleaning. A total of 20-plate type (65 × 10 × 3.3 mm) specimens for measuring surface roughness and flexural strength and 20-disc type (15 × 3 mm) specimens for measuring color and micro-hardness, were immersed in each cleaning solution. After mixing 75 mL of 2 mg/ml DM and 75 mL of 3% H_2_O_2_ to make 150 mL of DM, immersion was performed for a total of 12 days 16 h 10 min, reflecting 10 min a day for 5 years. DCT was made by foaming one tablet in 150 mL of lukewarm water, followed by immersion for a total 6 days 8 h 5 min, reflecting 5 min a day according to the manufacturer's instructions (Fig. 4). After the immersion treatment, the 3D printed denture base resins were washed with PBS and dried. Surface roughness, micro-hardness, and color were measured twice before and after cleaning, and the differences of the two values were calculated and compared. The flexural strength of denture base resin was measured after cleansing and compared with international standards for denture base resins.

The surface roughness of 3D printed denture base resin (plate type) was evaluated using a noncontact surface roughness tester (Contour GT-I; Bruker Inc.). The cut-off value was set at 0.8 mm with a speed of 0.5 mm/s. The difference in surface roughness (ΔR) was calculated as the arithmetic mean by measuring three distinct areas of the denture base resin. Values within 0.20 µm were considered clinically acceptable^30^. The micro-hardness of 3D printed denture base resin (disc type) was evaluated with a Vikers micro-hardness tester (MMT-X; Matsuzawa). Measurements were performed on each denture base resin with a Vikers diamond indenter under a load of 300 g for 15 s. The difference in micro-hardness (ΔHK) was calculated as the arithmetic mean by measuring three different parts of the denture base resin. The color change of 3D printed denture base resin (disc type) was evaluated with a spectrophotometer (CM-5 Spectrophotometer; Konica Minolta). The average L*, a*, and b* values measured in three different parts of the denture base resin were used to calculate the color change (Δ$${E}_{00})$$ using the following color difference formula (CIEDE2000).

$$\Delta E_{00}^{*} = \sqrt {\left( {\frac{{\Delta L^{\prime } }}{{k_{L} S_{L} }}} \right)^{2} + \left( {\frac{{\Delta C^{\prime } }}{{k_{C} S_{C} }}} \right)^{2} + \left( {\frac{{\Delta H^{\prime } }}{{k_{H} S_{H} }}} \right)^{2} + R_{T}\frac{{\Delta C^{\prime } }}{{k_{C} S_{C} }}\frac{{\Delta H^{\prime } }}{{k_{H} S_{H} }}^{2} }$$

The data were also quantified according to the National Bureau of Standards (NBS) units (NBS units = ΔE × 0.92) and the changes were classified as shown in Table 2. The flexural strength of 3D printed denture base resin (plate type) was determined by conducting a three-point bending test in accordance with ISO 20,795–1:2008 standard, using a universal testing machine (Instron 5942; Instron). With 50 mm between the two support spans and a crosshead speed of 5.0 mm/min, a force was applied to the center of the denture base resin until it fractured. Flexural strength was calculated by the formula below, using the peak load applied (*P*), span length (*L*), and specimen width (*b*) and thickness (*d*). Flexural strength values lower than 65 MPa were deemed clinically unacceptable.

**Table 2:** Classification of color change according to the NBS.

NBS unit	Definitions of color differences	
0.0–0.5	Trace	Extremely slight change
0.5–1.5	Slight	Slight change
1.5–3.0	Noticeable	Perceivable
3.0–6.0	Appreciable	Marked change
6.0–12.0	Much	Extremely marked change
12.0 or more	Very much	Change to another color

*NBS* National Bureau of Standards.

$$s(MPa)=\frac{3PL}{2b{d}^{2}}$$

Cytotoxicity testing was performed in accordance with ISO 10993-5. First, denture base resin was eluted in Dulbecco’s Modified Eagle’s Medium (LM 001-05; Welgene) containing 10% fetal bovine serum and 1% penicillin–streptomycin. An L-929 fibroblast cell suspension was prepared, and cells were counted using a hematocytometer. At a rate of 1 × 10^5^ cells/ml, the cell suspension was dispensed at 2 ml/well into each 6-well plate and cultured for 24 h. After removing the culture medium in each well and washing with Dulbecco's PBS (DPBS), the denture base resin and the solvent control were applied at 2 ml per well and placed in an incubator at 37 °C and 5% CO_2_ for 24 h. After incubation, the cells were carefully washed with DPBS, after which 2 ml of fresh medium was added. Then, CCK-8 solution (CCK-8; Dojindo Laboratories) was added, followed by incubation for 3 h, after which the absorbance was measured at 450 nm using a microplate reader (Infinite 200 Pro; Tecan). The number of cells was calculated by substituting for the standard, and the relative cell count (RCC, %) value was obtained by using the formula below. In accordance with the ISO 10993-5:2009(E) standard, an RCC exceeding 70% is considered to reflect an absence of cytotoxicity. Cell morphology was also observed using an optical microscope (CKX 41;Olympus) at 200 × magnification. Experimental and control groups for cytotoxicity test are shown in Table 3.

$${\text{RCC}}\;\left( \% \right) = \user2{ }\frac{{{\text{Cell }}\;{\text{number}}\;{\text{ of }}\;{\text{test}}\;{\text{ group }}}}{{{\text{Cell }}\;{\text{number }}\;{\text{of }}\;{\text{negative }}\;{\text{control}}}} \times 100$$

**Table 3:** Experimental and control groups for cytotoxicity test.

Groups	Solution	
Control group	Negative	Solvent control
Experimental group	DM at low concentration (2 mg/ml)	
	DM at high concentration (10 mg/ml)	
	DCT	
	3D printed denture base resin (no immersion)	

All statistical analyses were performed using SPSS for Windows (SPSS version 29; IBM Corporation). The distribution of the obtained data was evaluated for normality using the Shapiro–Wilk test. To compare the mean of each experimental group and the control group in the crystal violet staining experiment, one-way ANOVA followed by Scheffe’s post hoc test was performed. The data on surface roughness, color, micro-hardness, and flexural strength were analyzed by *t* test or Mann–Whitney U test depending on whether the data was normally distributed. p-values less than 0.05 were considered to reflect statistically significant differences.

## Results

**Figure 5:**
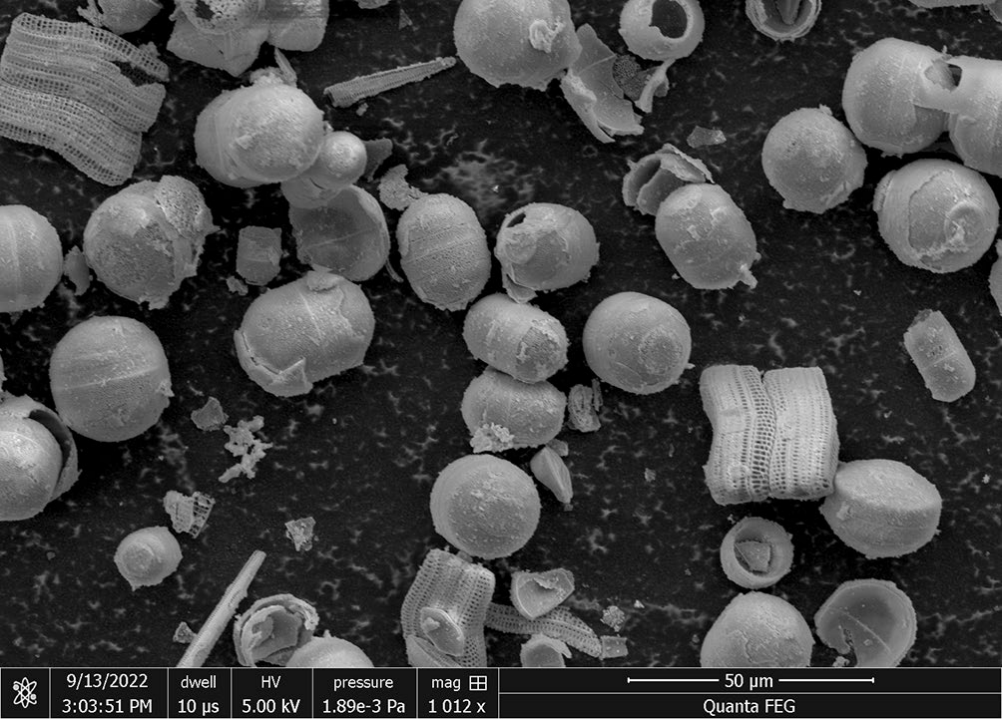
SEM image of MnO_2_-doped diatom complex *Melosira.*

MnO_2_-doped DM exhibited a spherical or ovoid shape, along with a porous structure. The average size of these particles was 20 µm and the average diameter of the pores of these particles was 500 nm (Fig. 5). The DM and DA, doped with MnO_2_, generated O_2_ microbubbles immediately after mixing with 3% H_2_O_2_. The generated O_2_ bubbles were continuously released from the inside of the hollow channels of the diatoms which moved in clusters with propulsion. As time passed, the generation of microbubbles decreased, and the propulsion speed also decreased (Supplemental Video Clip 1).

**Figure 6:**
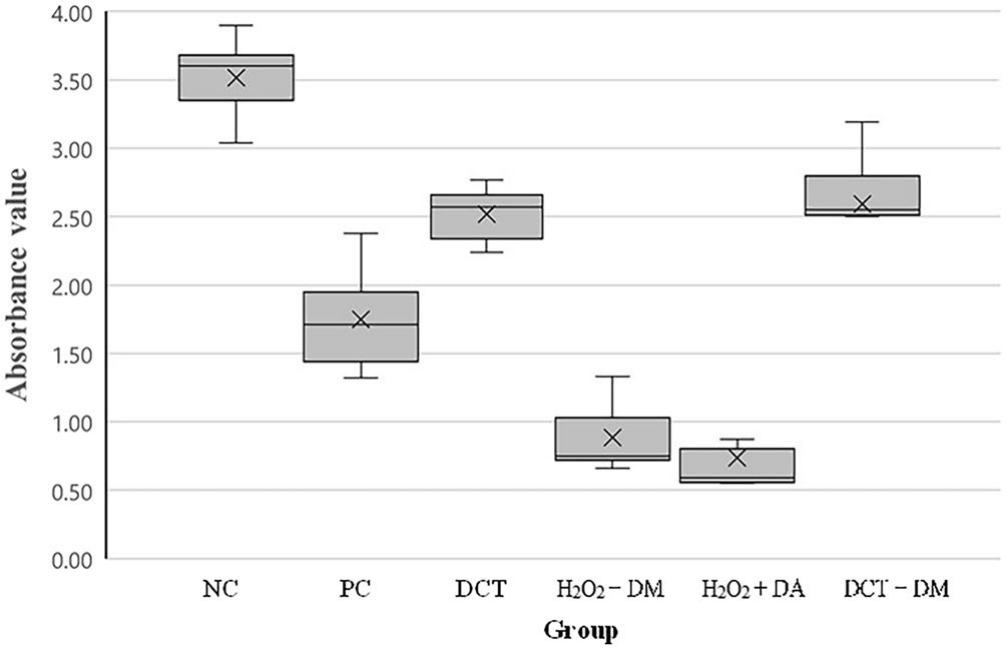
Boxplot of absorbance value after crystal violet staining. NC, negative control. PC, positive control. DCT, denture cleansing tablet. DM, diatom complex *Melosira*. DA, diatom complex *Aulacoseira*.

The average absorbance value after crystal violet staining was the lowest in the H_2_O_2_ with DA group at 0.737 ± 0.293, followed by the H_2_O_2_ with DM group at 0.885 ± 0.272. and then the PC group, DCT group, DCT with DM group, and NC group with values of 1.750 ± 0.399, 2.515 ± 0.220, 2.592 ± 0.436, and 3.513 ± 0.328, respectively (Fig. 6). The F value obtained using one-way ANOVA for the average value of the biofilm removal ability of the six groups was calculated to be 56.915, with a *p* value of < 0.001, indicating a statistically significant difference. Scheffe’s test was also conducted to evaluate the significance of the difference in mean values of each group through multiple comparisons. H_2_O_2_ with DA and H_2_O_2_ with DM had the lowest mean values, exhibiting the best biofilm removal abilities, with no significant difference between them. The average value of the PC group was the next lowest, followed by the DCT group and the DCT with DM group, with no statistically significant difference between these two groups. Finally, the NC group showed the lowest biofilm removal ability with the highest average value.

**Figure 7:**
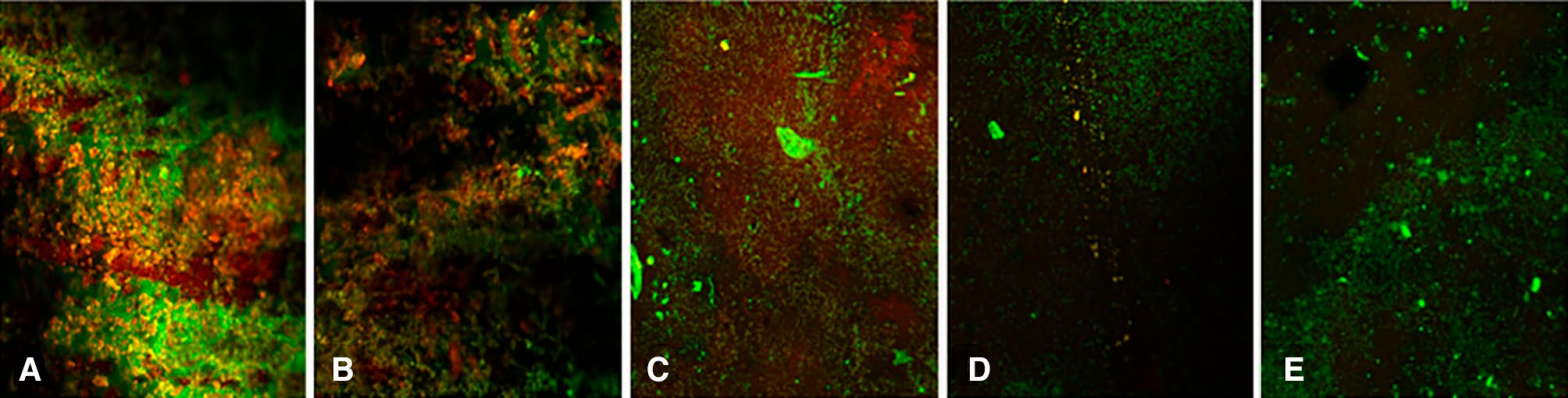
Confocal microscopy images of remaining biofilm after cleansing. Green showed living bacteria, and red showed extracellular polymeric substances. (**A**) negative control. (**B**) positive control. (**C**) denture cleansing tablet. (**D**) H_2_O_2_ with diatom complex *Melosira*. (**E**) denture cleansing tablet with diatom complex *Melosira*.

The effectiveness of biofilm removal was further confirmed through the analysis of residual EPS with immunostaining. The samples were observed under a confocal laser scanning microscope, after staining the bacterial cells with FITC (green) and EPS with ConA-TMR (red). NC group had the thickest biofilm, showing strong staining of both green and red. Both green and red were observed in the PC group and DCT group, but the biofilm was thicker in the PC group. In the H_2_O_2_ with DM group, weak green staining was observed, which indicated reduced bacteria presence. Although the absorbance result of the DCT with DM group was the second highest after the value of the NC group, only green fluorescence was observed in that group (Fig. 7).

The mean values of surface roughness of both DM and DCT groups, both before and after cleansing, were found to be within the clinically acceptable range of 0.2 µm. The ΔR of the DM group was -0.018 ± 0.081, while that of DCT group was -0.022 ± 0.064, showing a slight decrease in surface roughness in both groups, along with no statistically significant difference between the two groups (*p* = 0.904).

The mean ΔHK values of the DM and DCT groups were determined to be 0.461 ± 0.911 and -0.350 ± 0.864, respectively, indicating only slight changes in both groups. Furthermore, no significant difference was observed between the two groups (*p* = 0.056). Surface roughness and micro-hardness values before and after cleansing, and their change values are shown in Table 4.

**Table 4:** Measured values of surface roughness and micro-hardness.

Experimental group	Before		After		Change (Δ)	
	Mean ± SD	*P* value	Mean ± SD	*P* value	Mean ± SD	*P* value
Surface roughness ΔR (µm)						
DM	0.174 ± 0.058	0.970*	0.156 ± 0.069	0.791**	− 0.018 ± 0.081	0.904*
DCT	0.168 ± 0.047		0.146 ± 0.038		− 0.022 ± 0.064	
Micro-hardness ΔHK (HV)						
DM	11.732 ± 1.059	0.336*	12.193 ± 0.780	0.605*	0.461 ± 0.911	0.056*
DCT	12.321 ± 1.553		11.971 ± 1.086		− 0.350 ± 0.864	

*T test, **Mann–Whitney U test.

The mean ΔE values of the DM and DCT groups were determined to be 0.747 ± 0.331 and 0.902 ± 0.507, respectively. Calculating the NBS unit using ΔEoo, both groups showed a value below 1, indicating a ‘slight’ color change according to the NBS system. No statistically significant difference was identified between the two groups (*p* = 0.428). After the cleansing process, the mean values of flexural strength of the DM and DCT groups were determined to be 89.100 ± 10.472 and 86.702 ± 9.091, respectively. Both values exceeded the international standard of 65 MPa for denture base resin. Furthermore, there was no significant difference observed between two groups (*p* = 0.591). The results of statistical analysis are shown in Table 5.

**Table 5:** Change values of color and flexural strength.

Experimental group	Mean ± SD	*p* value
Color ΔE		
DM	0.747 ± 0.331	0.428
DCT	0.902 ± 0.507	
Flexural strength (MPa)		
DM	89.100 ± 10.472	0.591
DCT	86.702 ± 9.091	

**Figure 8:**
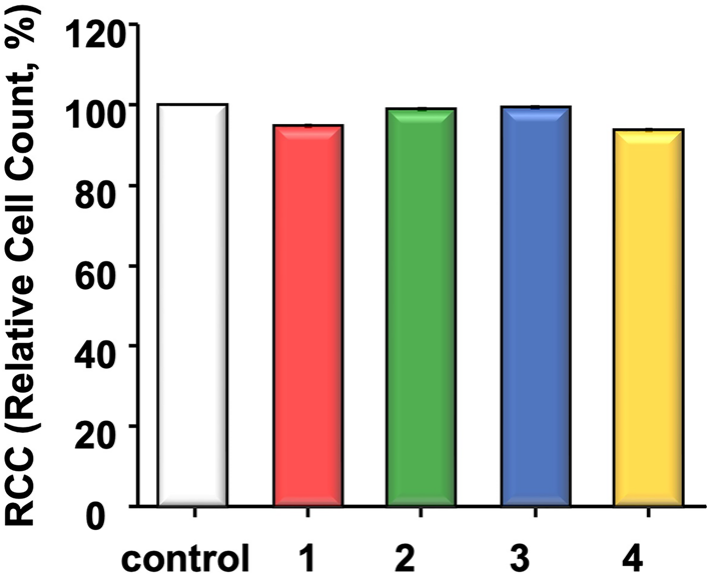
Relative cell count. 1, low concentration of diatom complex *Melosira*. 2, High concentration of diatom complex *Meloisra*. 3, denture cleansing tablet. 4, 3D printed denture base resin.

The RCC of all experimental groups was found to be 70% or higher, indicating the absence of cytotoxicity. Microscopic observation of cell morphology revealed no discernable differences between the control group and all the experimental groups (Fig. 8).

## Discussion

In this study, the biofilm removal ability of the diatom complex with active locomotion was evaluated and compared with the commercial denture cleanser and peracetic acid, which are used for disinfecting dental instruments. In addition, to assess the stability of the denture base material after long-term use of the diatom complex, physical and mechanical property tests and cytotoxicity tests were conducted before and after cleansing for five years. As a result, the DA and DM groups showed statistically significant higher biofilm removal ability than other groups, therefore the first null hypothesis that the biofilm removal ability of diatom complex is not different from that of dental cleanser tablet was rejected. As for the second hypothesis, DM had little effect on the roughness, hardness, shade, strength before and after cleaning, and cytotoxicity. Therefore, the hypothesis that stated that the diatom complex does not show any difference in the physical and mechanical properties and cytotoxicity of the denture base resin before and after cleaning, was accepted.

Diatoms are characterized by the formation of a porous biosilica frustule on the outside and have a high-dimensional structure with different pore size and distribution depending on the species^31^. The diatom complex doped with MnO_2_ react with H_2_O_2_ to generate microbubbles (O_2_) which are capable of self-locomotion and destroyal of the EPS. Our team evaluated the cleaning efficacy of DM on artificial saliva and compared it with the efficacy of DCT at various DM and H_2_O_2_ concentrations. As a result, 2 mg/mL DM + 3% H_2_O_2_ was found to have the best cleaning power, showing a cleaning effect comparable to that of the detergent^32^. Based on this, the final concentration to measure the cleaning ability of the diatom complex was determined.

DCT contains oxon, tetraacetylethylenediamine (TAED), sodium percarbonate, and sodium lauryl sulfate (SLS). The first step in the mechanism of the cleansing action is the dissolving of the two oxidizing agents to release H_2_O_2_, and to help remove stains. TAED then promotes the production of acetic acid peroxide and strengthens antibacterial action. Finally, SLS loosens the tartar and breaks the plaque matrix, lowering the surface tension^33^. Based on this mechanism, it was hypothesized that H_2_O_2_ released from DCT would enhance the biofilm removal capability when combined with DM, therefore this was established as an experimental group. The results showed that the DCT with DM group exhibited similar high absorbance values to the DCT group used alone. However, observation under microscopy after cleansing revealed that compared to DCT group, which showed both EPS and bacterial cells, only bacterial cells remained in the DCT with DM group. This might be that while DM disrupted the EPS protective layer through its reaction, it was unable to completely eliminate the remaining bacterial cells. It is possible that the amount of H_2_O_2_ released from DCT was insufficient or not released at all, which could have contributed to this result.

Peracetic acid is a disinfectant that acts as an oxidizing agent, destroying cell walls and oxidizing sulfur bonds in proteins or enzymes, thereby exhibiting rapid antimicrobial activity against a wide range of microorganisms^34^. Peracetic acid demonstrates good antimicrobial effects even when used alone. There is a synergistic effect when it is used as a mixture with hydrogen peroxide, which might be a result from weakening of the coat defensive barrier^35^. Based on this, the positive control group was established by mixing peracetic acid and H_2_O_2_ to compare with DM in biofilm removal ability. Additionally, the inner surface of the denture is curved rather than flat. Since plaque can easily attach to this inner surface, the specimen was designed in the form of a roof shape that imitates the intaglio surface of dentures, which are difficult to clean. Through observation of the angled inner surface of the specimen, it was confirmed that only a few bacterial cells remained with the treatment of DM group in comparison with the NC, PC, and DCT groups in which both bacterial cells and EPS were observed. DM, by destroying the EPS protective layer and effectively removing bacterial cells, can be described as having both mechanical and chemical cleansing effects. The DM is anticipated to address the issue where chemical cleansing agents were unable to impact the bacteria within the biofilm due to the protective layer of EPS.

Denture cleaners should not affect physical and mechanical properties of dentures such as discoloration, surface roughness, micro-hardness, and the strength while having the ability to remove inorganic deposits and stains^36^. Currently, denture fabrication using 3D printing is attracting attention from users because it is a simple, time and cost efficient method compared to the existing heat polymerization method. However, 3D printed resin is influenced by various factors such as build parameters, build orientation, post-curing process, number and thickness of layers, and interlayer shrinkage. These factors also affect the adhesion properties of the 3D printed resin^37^. If the internal structure consists of a microscopic space or defect with weak adhesion, the water absorption capacity increases, and denture materials immersed in denture cleaners can absorb more chemical cleaners, potentially altering their properties. According to a study, active oxygen generated during the foaming action of denture cleaner or the decomposition of H_2_O_2_ upon contact with water, may cause an increase in surface roughness due to the combination of mechanical action and high temperature^38^. The rough surfaces of dentures are more susceptible to microbial colonization, plaque build-up and discoloration. Therefore, the selected cleaning technique for dentures should be compatible with the denture base material to preserve its properties^39,40^.

To test this, we performed a simulation of a 5-year cleaning period using DM on 3D printed denture resin. We aimed to evaluate the impact of this cleaning period on the physical and mechanical properties of 3D printed denture resin and its effect on biocompatibility compared to DCT. There was a slight change in surface roughness after prolonged immersion in DM, but it remained within the clinically acceptable threshold of 0.2 μm. Similarly, there was a slight change in microhardness, but no significant difference was observed compared to DCT. Additionally, using the CIEDE2000 color difference formula, which has higher discriminatory power than conventional methods, both groups exhibited slight color changes with color difference values of less than 1 in NBS units, indicating a "slight" difference. The flexural strength of DM and DCT both demonstrated above 85 MPa after cleansing, surpassing the international standard specification for denture resins set by ISO 20,795-1, which is 65 MPa. However, a limitation of this study is that the denture cleanser and DM were not replaced every time for cleaning. DM and DCT demonstrate both mechanical and chemical mechanisms of action, however, the cleaning effect may be more related to the chemical action of the ingredients. After the foaming period, chemical components remaining in the solution may be responsible for changes in denture base material properties.

Finally, considering that dentures are used within the oral cavity, it was essential to evaluate the biocompatibility of DM. No significant cytotoxicity was observed after exposure to DM for 24 h at a concentration of up to 10 mg/mL, but cell viability was significantly reduced to 66% after exposure to 15 mg/mL. We evaluated cytotoxicity not only at low-concentration (2 mg/mL) of DM used in the experiment, but also at high-concentration (10 mg/mL) after long-term washing, and found that there was no cytotoxicity in all groups. DM is believed to be clinically usable as it has sufficient stability to be used as a denture cleaner. However, after washing with DM, it is necessary to rinse thoroughly with running water to remove residual chemicals and diatom particles. Additionally, since dentures are devices used in the oral cavity for long periods of time, additional research is needed that takes into account the invasion of bacteria and the effects of saliva.

Research in the field of dentistry using diatom complexes is still ongoing and there is a lack of prior studies focusing on oral biofilm removal. While assessing the biofilm removal capacity against a highly pathogenic organism with resistance to antimicrobial agents has been worthwhile, it is necessary to conduct further research to evaluate the efficacy of removing multi-species biofilms in an environment that closely resembles the oral cavity. With further research, DM can be expected to be commercialized as a natural new dental cleansing agent with both mechanical and chemical cleansing effects on dentures.

## Conclusions

Based on this study, the following conclusions were obtained.

1. The diatom complex made by doping MnO_2_ reacts with H_2_O_2_ to continuously generate fine air bubbles (O_2_), which is thought to reduce biofilm adhesion by destroying the EPS via self-propulsion and continuous bubble generation/rupture.
2. Long-term usage of the diatom complex on denture base resin results in minor changes on surface roughness, micro-hardness, flexural strength, and color, with no signs of cytotoxicity.

### Supplementary Information

Supplementary Legends.

Supplementary Video 1.

## Data Availability

The datasets used and/or analysed during the current study are available from the corresponding author on reasonable request.

## Abbreviations

EPS: Extracellular polymeric substances
NC: Negative control
PC: Positive control
DM: Diatom complex Melosira
DA: Diatom complex Aulacoseira
DCT: Denture cleanser tablet
DHCPs: Dental health care professionals
TSB: Tryptic Soy Broth
PBS: Phosphate-buffered saline
FITC: Fluorescein isothiocyanate
ConA-TMR: Concanavalin A, tetramethylrhodamine
NBS: National Bureau of Standards
DPBS: Dulbecco's PBS
RCC: Relative cell count
TAED: Tetraacetylethylenediamine
SLS: Sodium lauryl sulfate
