# Supplementary material for: Biofilm removal effect of diatom complex on 3D printed denture base resin
Source: Sci Rep. 2024 Feb 19;14:4034. doi: 10.1038/s41598-024-54408-y (PMC10874960; doi:10.1038/s41598-024-54408-y)
Supplement: Supplementary file 1 — Supplementary Legends. [file 41598_2024_54408_MOESM1_ESM.docx]

**Supplementary Video Legend**

Video clip1. Active micro-locomotion of MnO_2_-doped diatom in 3% H_2_O_2_ solution under an optical microscope (Leica S6D; Leica, Wetzlar, Germany)
